# Quantitative–Qualitative Discordance in Measuring Distress Among Intensive Care Unit Family Caregivers: A Mixed-Methods Study

**DOI:** 10.1177/26892820251408605

**Published:** 2026-02-10

**Authors:** Blair Wendlandt, Scott Vasher, Timothy Gee, Bradley Gaynes, Shannon Carson, Laura Hanson, Mark Toles

**Affiliations:** 1 Division of Pulmonary Diseases and Critical Care Medicine, Department of Medicine, University of North Carolina at Chapel Hill, Chapel Hill, North Carolina, USA.; 2 Section of Pulmonary and Critical Care, Department of Medicine, University of Chicago, Chicago, Illinois, USA.; 3 School of Medicine, University of North Carolina at Chapel Hill, Chapel Hill, North Carolina, USA.; 4 Department of Psychiatry, UNC School of Medicine, University of North Carolina at Chapel Hill, Chapel Hill, North Carolina, USA.; 5 Department of Epidemiology, Gillings School of Global Public Health, University of North Carolina at Chapel Hill, Chapel Hill, North Carolina, USA.; 6 Division of Geriatric Medicine and Palliative Care Program, Department of Medicine, University of North Carolina at Chapel Hill, Chapel Hill, North Carolina, USA.; 7 School of Nursing, University of North Carolina at Chapel Hill, Chapel Hill, North Carolina, USA.

**Keywords:** pain control, symptom assessment

## Abstract

**Background:**

The Impact of Events Scale-Revised (IES-R) is widely used in outcomes research involving family caregivers in the intensive care unit (ICU), but whether IES-R scores align with caregivers’ perceptions of their own distress is not known.

**Methods:**

We performed a secondary mixed-methods analysis, comparing 6-month IES-R scores and self-described wellness or distress outcomes in a semi-structured interview to categorize caregivers as experiencing quantitative–qualitative concordance or discordance and describing caregivers’ specific symptoms.

**Results:**

The frequency of quantitative–qualitative discordance was 4/18 (22%), and all cases involved a low IES-R score (suggesting quantitative wellness) and qualitative distress. These caregivers described physical and financial stress rather than psychological symptoms.

**Conclusion:**

One in four ICU family caregivers identified qualitative distress that was not captured by the IES-R. While the IES-R performed well in identifying caregivers with intense psychological symptoms, additional outcome measures are needed to identify caregivers experiencing physical or financial distress.

## Introduction

More than 1.5 million people are admitted to the intensive care unit (ICU) with acute cardiorespiratory failure each year in the United States, and the majority will become dependent on family caregivers following hospital discharge.^1–3^ These family caregivers are at risk of a range of negative long-term outcomes, referred to collectively as “psychosocial distress.”^4^ While a formally defined core outcomes set does not exist in this population, psychiatric symptoms such as anxiety, depression, and post-traumatic stress have been defined as research priorities by leading professional societies.^5^ Accordingly, instruments that measure psychiatric symptoms have become *de facto* core outcome measures. Originally developed to screen for symptoms suggestive of psychiatric diagnoses, these instruments are often applied as measures of generalized psychosocial wellness or distress in ICU family caregiver research,^4,6,7^ encompassing psychiatric symptoms as well as physical, social, and spiritual health.^8^ However, it is unclear whether the scores of the measures of psychiatric symptoms accurately portray ICU family caregivers’ experiences of psychosocial distress. We thus conducted this exploratory study to measure the frequency of discordance between the Impact of Events Scale-Revised (IES-R) measure of post-traumatic stress symptoms (PTSS) and qualitative assessments of generalized psychosocial distress and wellness among ICU family caregivers, and to describe the specific symptoms experienced by these caregivers. This *post hoc* analysis was developed to understand whether a scale designed to measure this psychiatric syndrome is aligned with ICU family caregivers’ self-described distress levels.^9,10^

## Methods

This convergent, parallel, mixed-methods analysis uses data from a completed prospective cohort study that performed both quantitative and qualitative assessments of distress for the family caregivers of adult patients admitted to the ICU with acute cardiorespiratory failure.^9–11^ In the parent study, quantitative assessments of caregiver distress were performed using the IES-R, a validated 22-question instrument designed to screen for clinically significant PTSS.^7^ Scores range from 0 to 88, and a score of 33 or more suggests clinically significant PTSS and a possible diagnosis of post-traumatic stress disorder. Following completion of the final quantitative assessment at 6 months after ICU admission (a widely used endpoint in critical care outcomes studies),^12^ caregivers were invited to participate in a semi-structured interview describing self-perceived wellness and distress. Further details about the coding framework and categorization of participant wellness and distress are described in the parent study.^10^

This mixed-methods analysis was guided by our adaptation of a previously established framework that defined core physical and psychological symptoms experienced by family caregivers of ICU survivors, such as depression, pain, and sleep problems.^13^ In this framework, both objective, symptom-based measurements and subjective, self-perceived health are important indicators of wellness.^14^ We undertook minor modifications to apply the framework to our study population, and the original interview transcripts were then re-coded to identify specific physical and psychological symptoms. We then examined the concordance of caregiver-reported IES-R scores measured at 6 months (quantitative data) and caregiver-reported experiences of wellness and distress (qualitative). For the quantitative assessment, caregivers with a 6-month IES-R score of ≥33 were categorized as quantitatively distressed, while caregivers with an IES-R score of <33 were categorized as nondistressed. A score of ≥33 has previously been validated as the cut-off for clinically significant PTSS in non-ICU populations and is widely used as the threshold for severe psychological distress in studies of ICU patients and family caregivers.^15^ For the qualitative assessment, caregivers were categorized based on their self-described outcome of either generalized wellness or distress. To integrate the quantitative and qualitative data, we described concordance in measures of caregiver-reported wellness and distress as indicated by the IES-R score and experiences of physical and psychological symptoms as indicated in the interview data. The findings were reported in a data matrix and narrative. All research activities were approved by the Institutional Review Board of the University of North Carolina at Chapel Hill (#20–0218).

## Results

Our analysis included 18 caregivers, which provided thematic saturation for the qualitative analysis. The participant characteristics are shown in Table 1. As indicated in Table 2, caregivers were categorized based on the concordance of the IES-R score and experiences of wellness and distress: (1) Quantitative–Qualitative Concordance, Wellness (IES-R score <33 and Qualitatively Well, N = 10); (2) Quantitative–Qualitative Concordance, Distressed (IES-R score ≥33 and Qualitatively Distressed, N = 4); (3) Quantitative–Qualitative Discordance, Wellness (IES-R score ≥33 and Qualitatively Well, N = 0); and (4) Quantitative–Qualitative Discordance, Distressed (IES-R score <33 and Qualitatively Distressed, N = 4). The overall frequency of Quantitative–Qualitative Discordance in this population was 4 of 18 (22%) cases, and all involved a low IES-R score and qualitative endorsement of distress. Caregivers experiencing Quantitative–Qualitative Concordance for distress described intense psychological symptoms, whereas caregivers experiencing Quantitative–Qualitative Discordance described symptoms of physical and financial stress. No discordance was noted among caregivers experiencing wellness, and these individuals described physical and psychological symptoms that were manageable or had resolved. The symptoms experienced by each group are summarized in Table 2.

**Table 1.** Participant Characteristics

Characteristic	Caregivers (*N* = 18)
Age, mean (SD)	56.6 (12.8)
Female, *n* (%)	12 (67)
Race	
White, *n* (%)	13 (72)
Black, *n* (%)	5 (28)
Relationship to patient	
Spouse, *n* (%)	11 (62)
Child, *n* (%)	4 (23)
Parent, *n* (%)	1 (5)
Sibling, *n* (%)	1 (5)
Grandchild, *n* (%)	1 (5)
Prior self-reported mental health diagnosis	
Depression, *n* (%)	5 (28)
Anxiety, *n* (%)	5 (28)
PTSD, *n* (%)	2 (11)
Patient deceased at 6 months, *n* (%)	7 (39)
Mean IES-R score at 6 months, mean (SD)	19.7 (21.6)
Qualitative outcome at 6 months	
Wellness, *n* (%)	10 (56%)
Distress, *n* (%)	8 (44%)
Time between quantitative and qualitative assessments, months (SD)	4.7 (2.5)

IES-R, Impact of Event Scale-Revised; PTSD, Post-traumatic stress disorder; SD, standard deviation.

**Table 2.** Core Symptoms and Exemplar Quotes

Mean IES-R score (SD), IES-R range	Qualitative outcome	Core symptoms and exemplar quotes
Quantitative–Qualitative concordance, wellness (*N* = 10)		
7.9 (6.8) 0–22	Wellness	Resolving grief: “I’m tryin’ not to cry. Today is nine months that he passed. The first two, three months, it was really hard on me, because we were together for 32 years and havin’ to lose someone that you were so used to seein’ every day and spendin’ time with, you love ‘im, and you have your family together. It was hard, so I joined a grief counseling group and that helped me through.” -Caregiver 074
		Manageable caregiving burden: “Well, I think because—that’s why I’ve said because of my mother’s health bein’ deteriorated, it’s actually—even though I am stressed, it’s, I don’t wanna say the new normal, but it’s just a process of doin’ what you have to do…”-Caregiver 067
Quantitative–Qualitative Concordance, Distress (*N* = 4)		
54.5 (13.7) 34–64	Distress	Depression/Anxiety: “I go into the depression. It puts me into depression. I have anxiety. I have panic attacks, something I never had when I was young. I never had that. I didn’t know what that was… but I know what it is now.”-Caregiver 070
		Persistent Grief: “In my life, I don’t feel that I have anybody to love me anymore. When you lose that, which I felt like I have, it’s not just the blow of the person not being here that hurts you so much. It’s the fact that you don’t have anybody to love you anymore. That’s gone. There’s nobody that will ever hug me or touch me or kiss me or hold me in that physical sense, smile at me in a knowing way because we could be in a room full of people and look across the room at each other, and you just get this feeling of security and knowing that you’re loved and you’re cared for and you’re cared about.”-Caregiver 066
Quantitative–Qualitative Discordance, Distress (*N* = 4)		
16.3 (12.8) 2–29	Distress	Physical Health Problems: “I eat a lot when I’m stressin’ a lot… I sleep a lot… but I think that’s a sign of the just tired and stressed and physically exhausted…I have gained a bunch of weight since all this, and that’s not good.”-Caregiver 069
		Financial Stress: “I about lost my bottom jaw a few weeks ago, though, ‘cause I got her Humana summary for the year… for her medical bills. The only medical bill my mom had was that encounter from the ICU and the hospital and everything. It was $1 million and $70,000... I know the hospital has to get paid somehow, but I don’t know that they need to send that on to the innocent caregiver here.”-Caregiver 099

## Discussion

No current set of long-term outcomes exists among ICU family caregivers; the current relevant outcomes were chosen over a decade ago at a stakeholder conference consisting entirely of medical professionals.^5^ In this exploratory study, we identified a vulnerable subset of family caregivers that experienced sustained psychosocial distress that would be overlooked under the current research measurement paradigm. While qualitative wellness was consistently associated with a low IES-R score in our sample, we found that half of the caregivers who endorsed qualitative distress were mis-represented by a low IES-R score. Our results suggest that the IES-R functions reasonably well as an indicator of psychosocial distress characterized by psychological symptoms, as it was designed to do, but may fail to capture distress characterized by other important long-term impacts, such as physical discomfort or financial strain. The existing guidance for palliative care clinicians who provide consultation in the ICU focuses on strategies to ameliorate post-traumatic stress and other psychological symptoms for family caregivers. Our findings suggest that many caregivers experience unrecognized suffering in other domains, such as physical and financial health, and that support strategies may need to be tailored accordingly if we want to educate, prepare, and assist these caregivers and the patients under their care.^16^

Our study has several limitations, including a small sample size and the asynchronous collection of quantitative and qualitative assessments. We did not find significant differences in the interval between the two assessments, with 3.5 months for discordant caregivers versus 5.0 months for concordant caregivers (*p* = 0.3), and previous studies including serial assessments of PTSD symptoms have shown that the large majority of participants have stable symptom levels.^17^ Our study also has several strengths, including a novel research question that serves as a starting point toward advancing the science of psychosocial support interventions for ICU family caregivers. Future work should focus on developing a comprehensive core long-term outcomes set, developed in partnership with family caregivers, to ensure that research studies are focusing on the outcomes that are important to these caregivers and are reflective of their self-perceived experiences of psychosocial distress and wellness.

## Abbreviations

ICU: intensive care unit
PTSS: post-traumatic stress symptoms
IES-R: Impact of Event Scale-Revised
